# NEONATAL MORBIDITY NEAR MISS IN TERTIARY HOSPITALS IN A CAPITAL OF NORTHEAST BRAZIL

**DOI:** 10.1590/1984-0462/;2019;37;3;00011

**Published:** 2019-07-04

**Authors:** Danyelle Rodrigues Pinheiro de Araujo Brasil, Mirella Bezerra Rodrigues Vilela, Karla Eveline Ximenes de França, Silvia Wanick Sarinho

**Affiliations:** a Universidade Federal de Pernambuco, Recife, PE, Brazil.

**Keywords:** Near miss, healthcare, Morbidity, Early neonatal mortality, Infant, newborn, Health care, Assistance, Near miss, Morbidade, Mortalidade neonatal precoce, Recém-nascido, Atenção à saúde, Assistência

## Abstract

**Objective:**

To characterize near miss neonatal morbidity in tertiary hospitals in a capital city of Northeast Brazil based on Health Information Systems, and to identify differences regarding indicators of near miss cases, allowing the surveillance of newborns with risk of death.

**Methods:**

A cross-sectional study carried out in hospitals with neonatal intensive care unit, whose neonatal near miss cases in 2012 were identified from a deterministic linkage between the Mortality Information System and the Live Birth Information System. The biological variables of children, variables related to maternal characteristics and indicators of near miss were calculated by type of service and hospital. Biological variables of children, variables related to maternal characteristics and near miss indicators were calculated by service type and hospital and then compared by ratio difference test, parametric and non-parametric tests for measures of central tendency.

**Results:**

Of 24,254 live births, 2,098 cases of neonatal morbidity *near miss* were identified, most of them concentrated in the public hospitals (89.9%). The combination of birth weight and gestational age had the largest number of cases in both segments, public (43.5%) and private (46%). Variations in neonatal near miss indicators were observed between hospitals, which suggests assistance problems.

**Conclusions:**

The concept of neonatal near miss, its applicability with data from Health Information Systems, and its indicators are a preliminary tool to monitor hospital care for newborns by signaling health services that require in-depth evaluation and investments for quality improvement.

## RESUMO

**Objetivo:**

Caracterizar a morbidade neonatal *near miss* em hospitais terciários de uma capital do Nordeste do Brasil, por meio dos Sistemas de Informação em Saúde, e identificar diferenças quanto aos indicadores de *near miss* que possibilitem a vigilância dos recém-nascidos com risco de morte.

**Métodos:**

Estudo transversal realizado em hospitais com unidade de terapia intensiva neonatal cuja população foram os casos de *near miss* neonatal nascidos em 2012, identificados mediante o *linkage* determinístico entre os bancos de dados do Sistema de Informações sobre Mortalidade e do Sistema de Informações sobre Nascidos Vivos. As variáveis biológicas das crianças, as referentes às características maternas e os indicadores de *near miss* foram calculados por tipo de serviço e por hospital e comparados por meio de testes de diferença de proporção e testes paramétricos e não paramétricos para medidas de tendência central.

**Resultados:**

Do total de 24.254 nascidos vivos, foram identificados 2.098 casos de morbidade neonatal *near miss*, com a maioria no segmento público (89,9%). O peso ao nascer agregado à idade gestacional concentrou o maior número de casos em ambos os segmentos, público (43,5%) e privado (46%). Foram observadas variações nos indicadores de *near miss* neonatal entre os hospitais, sugerindo problemas assistenciais.

**Conclusões:**

O conceito de *near miss* neonatal, sua aplicabilidade com base nos dados dos Sistemas de Informação em Saúde e seus indicadores constituem uma ferramenta preliminar para monitorar a assistência hospitalar ao recém-nascido ao sinalizar serviços de saúde que necessitam de investigação aprofundada e investimentos para a melhoria da qualidade.

## INTRODUCTION

The concept of near miss has been used in the area of maternal health as a tool to assess and improve quality of care.^1^ By analogy, newborns with markers of severity at birth but who survive the neonatal period are considered neonatal near-miss cases.^2^ Brazil has been conducting studies on neonatal near miss in Latin America, but there is no international consensus on a standard definition for such cases.^3^ ^,^ ^4^

Recent research indicates that applying this concept to the neonatal context, just like to maternal context, can be useful to improve the quality of care for newborns and to identify failures in health services aimed at these clients.^5^ ^,^ ^6^ ^,^ ^7^ ^,^ ^8^ As the number of survivals is about three to six times greater than the number of deaths,^8^ monitoring neonatal care based on this concept in places with low neonatal mortality rate would be an advantage, and could provide more data for investigations and greater acceptance by health teams to discuss morbidity rather than mortality.^9^ ^,^ ^10^

Some studies have proposed indicators for this phenomenon based on markers used in the neonatal death risk prediction model: ^5^ ^,^ ^6^ ^,^ ^8^ ^,^ ^11^ newborn and gestational variables (pragmatic criteria) and markers used as substitutes for organic disorders or any disorder related to clinical management (management criteria). However, some questions regarding near miss neonatal morbidity predate the establishment of criteria and are still pending answers. Inference of the quality of neonatal care, one of the main potentialities of the concept, is based on empirical studies^5^ ^,^ ^6^ ^,^ ^8^ ^,^ ^11^ that do not cover important aspects of service evaluation from the perspective of health teams, for example.^12^ ^,^ ^13^

The applicability of the near-miss concept to different scenarios is also a major challenge. Attention should be paid to the evaluation of neonatal near miss in different health system contexts, by observing patients’ trend of and services’ characteristics. Pragmatic criteria collected retrospectively is recommended to define near miss neonatal cases whenever possible.^6^ In places with more resources available, a combination of pragmatic and managerial criteria can be used in prospective studies.^3^ In addition, there are differences as to socio-demographic and organizational characteristics of health services that require a more in-depth analysis.^10^ ^,^ ^14^

An alternative to facilitate the application of neonatal near miss concept is to use Health Information Systems (HIS).^15^ Some gestation and newborn variables (birth weight, gestational age and 5-minute Apgar score) are gathered from routine use official systems, such as the Information System on Live Births (Sinasc). These variables are used in health research in Brazil and have a good level of adequacy.^16^ ^,^ ^17^ We then raise the possibility of using data from official databases to establish indicators for neonatal near miss, with possible systematic use in large scale at health services, in order to compare services or a single service over time.

This article aims to describe neonatal near miss cases in a capital of Northeast Brazil based on HIS data and to identify preliminary warning differential aspects related to it at neonatal care services that receive patients at risk of neonatal death.

## METHOD

This is a cross-sectional study based on data from Sinasc and the Mortality Information System (MIS) from eight hospitals (four public and four private) that provided intensive neonatal care in Recife in 2012. The institutions selected had high-tech care services, adult and neonatal Intensive Care Units (ICU), and which, therefore, concentrated the largest number of high-risk births.

The study population was composed of neonatal near miss cases identified according to previously validated criteria: five newborns who presented any risk condition at birth (5th minute Apgar <7, weight <1,750 g or gestational age <33 weeks) and survived until the 7^th^ day of life. All neonates who were born in 2012 at the hospitals selected for the study were analyzed.

The variables related to newborns (gender, birth weight, 5^th^ minute Apgar score, gestational age, type of delivery and early neonatal death) and to mothers (age, parity, pre-natal care, type of gestation and place of birth) were used to characterize neonatal near miss cases per type of service.

Hospitals chosen for the survey had 24,254 births and 460 early neonatal deaths recorded in Sinasc and MIS. By deterministic linkage between databases, using as search means the number of live birth declaration included in death certificates, confirmed by the mother’s name, 2,098 cases of neonatal near miss were identified. Cases were analyzed according to risk criteria and type of health service (public and private) as a means of preliminarily contrasting possible differences between services as to the characteristics of the population assisted. Proportional differences were tested using Pearson’s chi-square test or the binomial test, both set with α=5%. Neonatal near miss indicators^6^ and the rate of early neonatal mortality were calculated to characterize neonatal near miss cases and then compare them between services:

- Neonatal near miss case: survival to the 7th day of life and presenting a risk condition at birth (5^th^ minute Apgar <7, birth weight <1,750g, or gestational age <33 weeks).
- Neonatal near miss rate (NNMR): number of neonatal near miss cases divided by the total number of live births multiplied by one thousand.
- Severe neonatal outcome rate (SNOR): number of neonatal near miss cases plus early neonatal deaths divided by the total number of live births multiplied by one thousand.
- Early neonatal mortality index (ENMI): number of newborn deaths in the first week of life when presenting life-threatening conditions at birth divided by the total number of newborns with life-threatening conditions at birth multiplied by 100.
- Early neonatal mortality rate (ENMR): number of early neonatal deaths divided by the total number of live births multiplied by one thousand.

Services were compared using the cutoff point corresponding to the median of prevalence indicators.

The distribution of indicators was tested with the Shapiro-Wilk test, with α=5%. Central tendency and dispersion were measured and the differences in values of indicators between the eight hospitals studied were determined by the Student’s t-test for independent samples if normal distribution, and by the Wilcoxon test for independent samples whenever indicated, both set at α=5%.

The study was approved by the Research Ethics Committee, under the Certificate of Presentation for Ethical Approval (CAAE) number 24756813.5.0000.5192.

## RESULTS

Of the 24,254 live births in 2012 at the hospitals selected, 2,098 neonatal near miss cases were identified, making up 4,6 cases for each neonatal death. From the total, 89.9% of neonates were born at public hospitals, and despite the discrepancy in absolute number of births when comparing public and private services, there was no statistically significant difference (p>0.05) between cases added based on the neonatal near miss criteria (Table 1).

**Table 1:** Criteria for selecting neonatal near miss according to health service system: public and private. Recife, 2012.

	Health system				Total		p-value
	Public		Private				
	n	%	n	%	n	%	
Number of cases	1,887	89.9%	211	10.1	2,098	100	
*Near miss* cases selected only by 5th-minute Apgar <7	188	10.0	25	11.8	213	10.2	0.39*
*Near miss* cases selected only by GA <33 weeks	77	4.1	10	4.7	87	4.1	0.70*
*Near miss* cases selected only by birth weight <1,750 g	332	17.6	36	17.1	368	17.5	0.90*
*Near miss* cases selected by birth weight < 1,750 g and 5th-minute Apgar <7	18	1.0	1	0.5	19	0.9	0.48*
*Near miss* cases selected by birth weight < 1,750 g and GA <33 weeks	821	43.5	97	46.0	918	43.8	0.49*
*Near miss* cases selected by 5th-minute Apgar <7 and GA <33 weeks	77	4.1	10	4.7	87	4.1	0.65*
*Near miss* cases selected by all three criteria	81	4.3	4	1.9	85	4.1	0.05*

GA: gestational age; *binomial test for two proportions.

The comparison between health service networks showed that birth weight was the single criterion with the largest number of cases in both segments, followed by 5th minute Apgar and gestational age. As for newborns presenting two or three risk criteria, birth weight and gestational age were linked to the largest number of cases in both segments (Table 1).

As for maternal and prenatal characteristics, there were statistically significant differences in maternal age, lack of or inadequate prenatal care, mostly at public hospitals (Table 2).

**Table 2:** Characterization of near miss neonatal cases per maternal and prenatal data according to health service system: public and private. Recife. 2012.

		Public		Private		Total		p-value*
		n	%	n	%	n	%	
Total		1.887		211		2.098		
Mother’s age	10 a 19	256	13.6	4	1.9	260	12.4	<0.001
	20-35	1.542	81.7	188	89.1	1.730	82.5	
	36 or older	89	4.7	19	9.0	108	5.1	
Type of pregnancy	Single	1.657	87.8	159	75.4	1.816	86.6	<0.001
	Twin	215	11.4	49	23.2	264	12.6	
	3+	15	0.8	3	1.4	18	0.8	
Prenatal care**	0 a 3	537	29.6	7	3.4	544	26.9	<0.001
	4 a 6	898	49.5	36	17.6	934	46.3	
	7 +	379	20.9	162	79.0	541	26.8	
Parity	Multiparous	479	25.4	60	28.4	539	25.7	0.05
	Primiparous	1.102	58.4	130	61.6	1.232	58.7	
	Multiparous	306	16.2	21	10.0	327	15.6	

*Pearson’s χ^2^ test; ** 73 cases were excluded from the analysis in the public segment and six cases in the private segment whose variable information about the number of visits was ignored.

As for biological and birth variables, there were statistically significant differences as for type of delivery, with emphasis on the private system, in which 93.3% of near miss cases were of neonates born by cesarean section (Table 3).

**Table 3:** Characteristics of neonatal near miss cases as for biological and birth variables according to health service system: public and private. Recife. 2012.

		Public		Private		Total		p-value*
		n	%	n	%	n	%	
Total		1.887		211		2.098		
Sex**	Female	928	49.2	107	50.7	1.035	49.3	0.82
	Male	957	50.8	104	49.3	1.061	50.7	
Delivery***	Vaginal	881	46.8	14	6.7	895	42.7	<0.001
	C-section	1.003	53.2	196	93.3	1.199	57.3	
Duration of gestation****	<33 weeks	1.346	71.5	149	71.3	1.495	71.5	0.30
	≥33 weeks	536	28.5	60	28.7	596	28.5	
Birth weight	<1.750 g	1.609	85.3	176	83.4	1.785	85.1	0.54
	≥1.750 g	278	14.7	35	16.6	313	14.9	
5th-minute Apgar*****	<7	286	15.4	30	14.2	316	15.3	0.55
	≥7	1.575	84.6	181	85.8	1.756	84.7	

*Pearson’s χ^2^ test; **two cases of the public segment whose information about the variable sex was ignored were excluded from the analysis; ***three cases of the public system were excluded from the analysis. and one case of the private system whose variable information was ignored; ****Five cases were excluded from the analysis in the public segment and two cases in the private segment. whose variable information was ignored; *****26 cases were excluded from the analysis in the public segment, as the information about this variable was ignored.

Variations in neonatal near miss indicators between health services were found to be statistically significant. Using the median values to establish cutoff points for prevalence indicators, hospitals with NNMR above 67.1/thousand live births and SNOR above 76.3/thousand live births presented a mortality index varying from 10.4 to 18.3%. Hospitals with prevalence indicators below the aforementioned cutoff points had mortality rates ranging from 2.3 to 12.9% (Table 4).

**Table 4:** Indicators of neonatal mortality and neonatal near miss morbidity per hospital and health service system: public and private. Recife. 2012.

Service	Hospitals from SUS				Public Subtotal	Private hospitals				Private subtotal	Total	p-value	Total descriptive analysis		
	A	B	C	D		E	F	G	H				Median	Q1	Q3
Near-miss cases	768	450	453	216	1.887	47	61	42	61	211	2.098				
LB per institution	6.435	4.179	4.019	2.097	16.730	1.508	2.046	1.562	2.407	7.523	24.253				
ENMR (per 1.000 LB)	36.7	25.6	14.2	15.7	25.8	2.6	3.9	0.6	5.8	3.6	19.0	0.02*	10.0	3.6	18.2
NNMR (per 1.000 LB)	119.3	107.7	112.7	103	112.8	31.2	29.8	26.9	25.3	28.0	86.5	0.007**	67.1	29.1	108.9
SNOR (per 1.000 LB)	156	133.8	126.9	118.7	138.5	33.8	33.7	27.5	31.2	31.6	105.5	0.007**	76.3	33.1	128.6
ENMI (%)	18.3	16.4	10.4	12.4	15.4	4.1	12.9	2.3	12.9	9.0	14.8	0.001**	12.7	8.8	13.8

SUS: Brazilian Public Health System; LB: live births; ENMR: early neonatal mortality rate; NNMR: neonatal near miss rate; SNOR: severe neonatal outcome rate; ENMI: early neonatal mortality index; *Student t-test for independent samples; ** Wilcoxon test for independent samples.

## DISCUSSION

According to the classification criteria applied,^5^ 4.6 cases of neonatal near miss were identified for each neonatal death, a value close to those verified in Brazilian studies on the concept.^5^ ^,^ ^8^ ^,^ ^18^ This result may be related to research sites: hospitals of the capital with greater technological complexity and quantitative of severe cases, presenting a higher neonatal near miss morbidity rate.

The distribution of neonatal near miss cases contributed to drawing a profile of the phenomenon at the services selected for the study. The public network had the largest number of neonatal near miss cases when composed of the three associated criteria, a fact already described in the literature,^19^ since most newborns in more severe conditions are from the poorest economic classes and often assisted at public hospitals.

Regarding the selection of neonatal near miss entry criteria, birth weight <1,750g was indicated as the isolated variable that concentrated the largest number of cases, corroborating with studies that associate low weight with higher risk of death in the neonatal period.^14^ ^,^ ^20^ 5th minute Apgar <7 concentrated the second highest percentage isolated for both segments. Intrapartum asphyxia is a preventable neonatal death cause that requires specific actions. In Brazil, the reduction of this death cause is closely associated with hospital care at birth and to birth itself, since the absolute majority of deliveries occur in hospital setting and are supported by qualified professionals.^20^ ^,^ ^21^

The prevalence of maternal age between 20 and 35 years indicates a higher survival of newborns with mothers in this age range, which corroborates previous studies that show reproductive extremes offering more risk of mortality to both the mother and the baby.^22^ ^,^ ^23^ When the public and private networks were analyzed separately, the former was found to have a sevenfold proportion of mothers aging between 10 and 19 years compared to the private network. The literature indicates that pregnant adolescents have lower family income, less attention during prenatal care, and many do not have a partner,^22^ which brings about a reflection on the difficulties of access to reproductive health care and on the quality of educative and health actions aimed at adolescents by the Brazilian Public Health System (SUS).

As for prenatal care visits, almost half of women included in sample had been to four to six consultations, and the private segment had the largest number of mothers who attended seven or more consultations. This better result in private hospitals, however, does not seem to reflect the number of operative deliveries, much higher than recommended by the World health Organization (WHO). It is still valid to emphasize that the evaluation of the number of prenatal visits alone does not necessarily result in better care or better perinatal outcome.^14^

The high proportion of caesarean section deliveries occurred in 57.3% of cases. The literature has shown that children born by cesarean section are more encompassed by neonatal near miss morbidity rate than those born by vaginal delivery.^8^ ^,^ ^23^ It is possible that therapeutic cesarean sections may have occurred in cases of maternal diseases to prevent stillbirths, but one must consider that iatrogenic cesarean section, often associated with preterm birth, increased neonatal respiratory morbidity, hospitalizations in the ICU and need of mechanical ventilation, is probably responsible for some cases of neonatal near miss.^8^ ^,^ ^23^ This should be better elucidated, especially in the private network, where there is a high proportion of operative deliveries. In this study, the percentage of the private segment was 93.3%, similar to the worrying rate of cesarean sections in Brazil’s supplementary network.^14^

The large number of absolute or proportional neonatal near miss (translated by the NNMR indicator) is an alert to the risky condition of patients who seek the health service, also showing the need to verify, at least in quantitative terms, the severe outcomes (translated by the SNOR indicator) for each hospital. In this study, NNMR and SNOR had higher values at hospitals of SUS, but some services with values differing for prevalence indicators had fatalities (similar ENMI). Although these indicators do not consider important aspects that can distinguish services, such as the availability of neonatal ICU beds or access to health care in more complex hospitals, they were able to show preliminary differences between health facilities pointing to possible differences in neonatal care.

The interpretation of findings regarding the prevalence of neonatal near miss cases and their significance to improve clinical care to newborns has not yet solved gap: there is no consensus on the generalization of the neonatal near miss concept, which can be compared in several contexts to guide the creation of interventions for each severe neonatal morbidity condition.^2^ ^,^ ^4^ ^,^ ^11^ There is a tendency to aggregate markers of organ dysfunction or clinical management, but no consensus on the most appropriate variables has been established.^3^ ^,^ ^4^

Some studies have established and tested neonatal near miss indicators, including common variables with different cutoff points, such as birth weight, 5th minutes Apgar score and gestational age, but with divergences in clinical variables indicating severity, such as mechanical ventilation, presence of congenital and other malformations, besides assessing survival to different neonatal periods (early neonatal period or entire neonatal period).^5^ ^,^ ^6^ ^,^ ^7^ ^,^ ^8^ ^,^ ^18^ In general, these studies had good accuracy in detecting neonatal near miss cases.

One of the first studies on neonatal near miss in Brazil pointed the prevalence of this phenomenon as possibly interpreted in the light of other indicators besides NNMR, such as SNOR and ENMI.^6^ Its use in the identification of remediable factors of the health system for the improvement of care for newborns has been reported. The aim of the current study, however, was to make the use of the indicator as a preliminary mode of neonatal care surveillance possible. Its application as indicator of service quality, adding variables related to severity criteria for newborns and others that reflect the quality of the service, seems to be useful for a more detailed prospective evaluation at institutions with more resources.^3^ ^,^ ^18^

This study used the variables gestational age <33 weeks, birth weight <1.750g and 5th minute Apgar <7, which showed adequate sensitivity and specificity and have been previously validated in Brazil.^5^ A recent study found that the definition of neonatal near miss exclusively by pragmatic criteria is valid and can be used to monitor neonatal severe morbidity.^18^ The methodological option is justified in the study, since the aim was to apply the indicator for sentinel surveillance of severe morbidity and to allow preliminary comparisons between health units and within the same unit over time in a more feasible way, based on retrospective data that are easily obtainable from HIS.

Only establishments with high technological complexity were selected in the study, and the application of the indicator was restricted to this type of health service because it concentrates a higher rate of severe cases. The scientific literature questions whether ICU services should use neonatal near miss criteria by adding complex indicators of severity such as the Clinical Risk Index for Babies (CRIB) and the Score for Neonatal Acute Physiology and Perinatal Extension (SNAPPE).^24^ ^,^ ^25^ However, once it requires laboratory support, it is more difficult to put to practice. The selection of exclusively pragmatic criteria for this study was also based on this justification. In addition, the difficulty of access to health care services may interfere with the survival rate of newborns.^14^ For this reason, only neonatal near miss cases involving birth and survival to early neonatal period at the same establishment were analyzed.

Despite the relevance of results found here, the authors recognize that there are limitations. This analysis is restricted to the first week of life of newborns, and a newborn survival in this period does not mean that they will survive the whole neonatal period. This short period was selected because of the higher concentration of early neonatal deaths^26^ and also to improve the application of the neonatal near miss concept and its indicators.

In addition, there is a need to recognize that differences in indicators between institutions should not lead to the simplistic conclusions that higher rates are found in institutions with greater problems in neonatal care, given the complex causal chain involving early neonatal deaths and considering that the population in the condition of interest has heterogeneous severity.^8^ Moreover, in this study, severity of cases of neonatal morbidity were not researched, neither was the quality of care provided by each health institution, However, the variance of indicators signals the possibility of problems related to hospital care and, thus, an opportunity to establish priorities when an on-site evaluation is necessary.

Finally, we conclude that identifying neonatal near miss cases and their indicators reopens the discussion on the concept of sentinel event for health surveillance by signaling the need for investigations of such cases, which allows pinpointing and evaluating the full set of maternal and child care, from family planning/prenatal care to hospital care for pregnant women and the newborn.

It is also worth mentioning that, despite the limitations, the criterion used as preliminary indicator has discriminatory power, and its applicability based on data resulting from the linkage between MIS and Sinasc databases supports its operationalization.
